# Supplementary material for: Inhibition of Checkpoint Kinase 1 (CHK1) Upregulates Interferon Regulatory Factor 1 (IRF1) to Promote Apoptosis and Activate Anti-Tumor Immunity via MICA in Hepatocellular Carcinoma (HCC)
Source: Cancers (Basel). 2023 Jan 30;15(3):850. doi: 10.3390/cancers15030850 (PMC9913340; doi:10.3390/cancers15030850)
Supplement: Supplementary file 1 [file cancers-15-00850-s001.zip › Supplementary Figure S1.pdf]

Supplementary Figure S1 Uncropped western blotting gels.

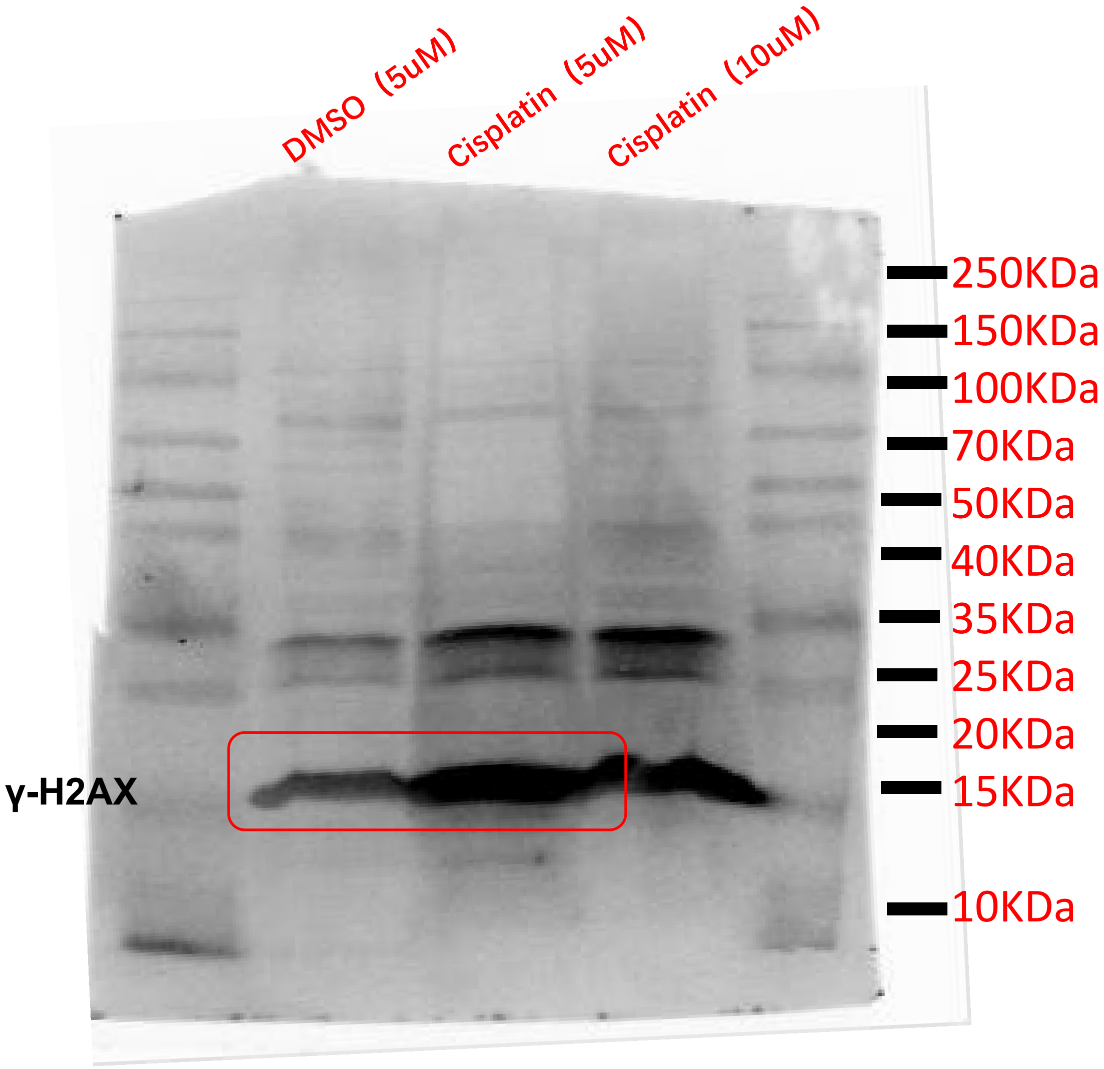

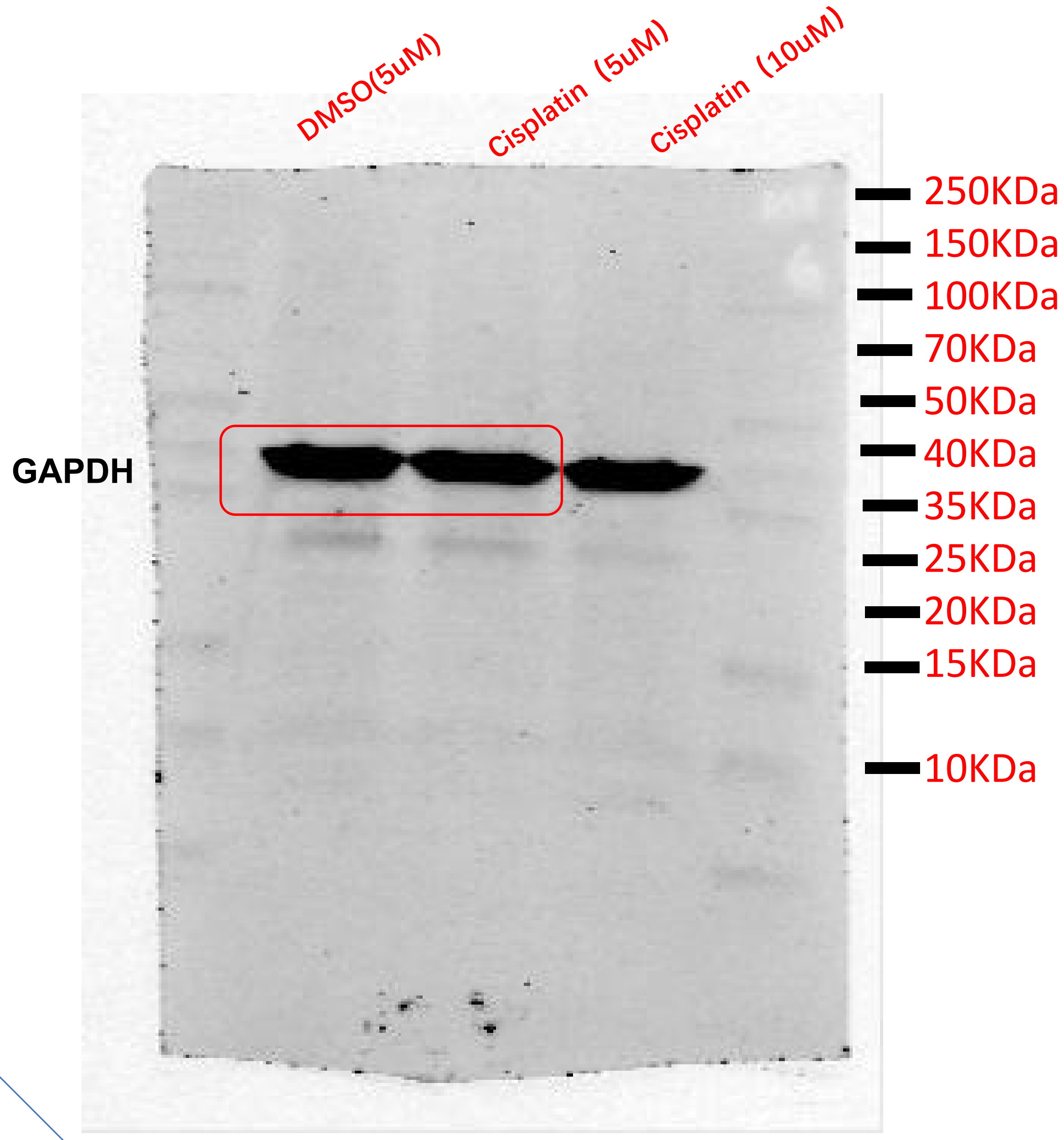

Fig.2a Huh-7 γ-H2AX and GAPDH

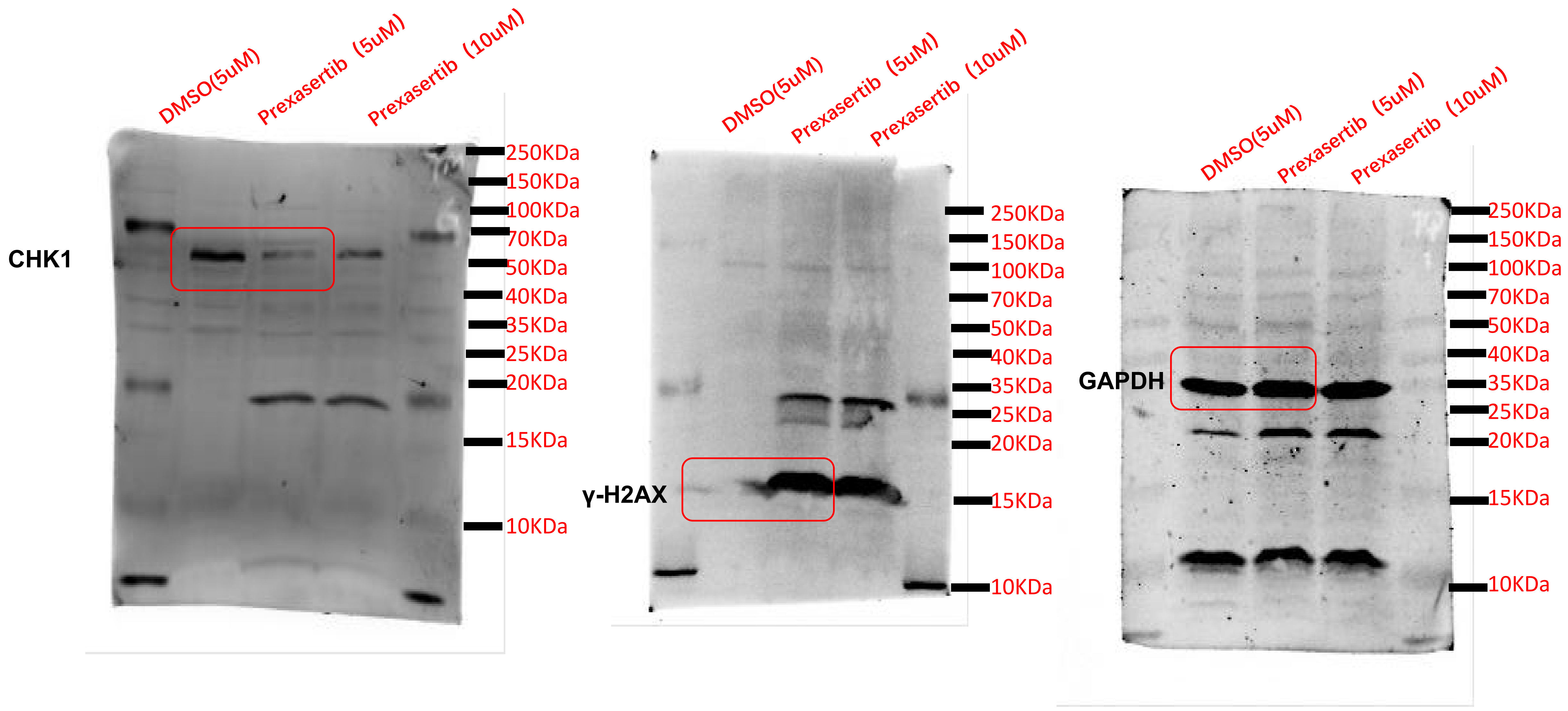

Fig.2b HepG2 CHK1, γ-H2AX and GAPDH

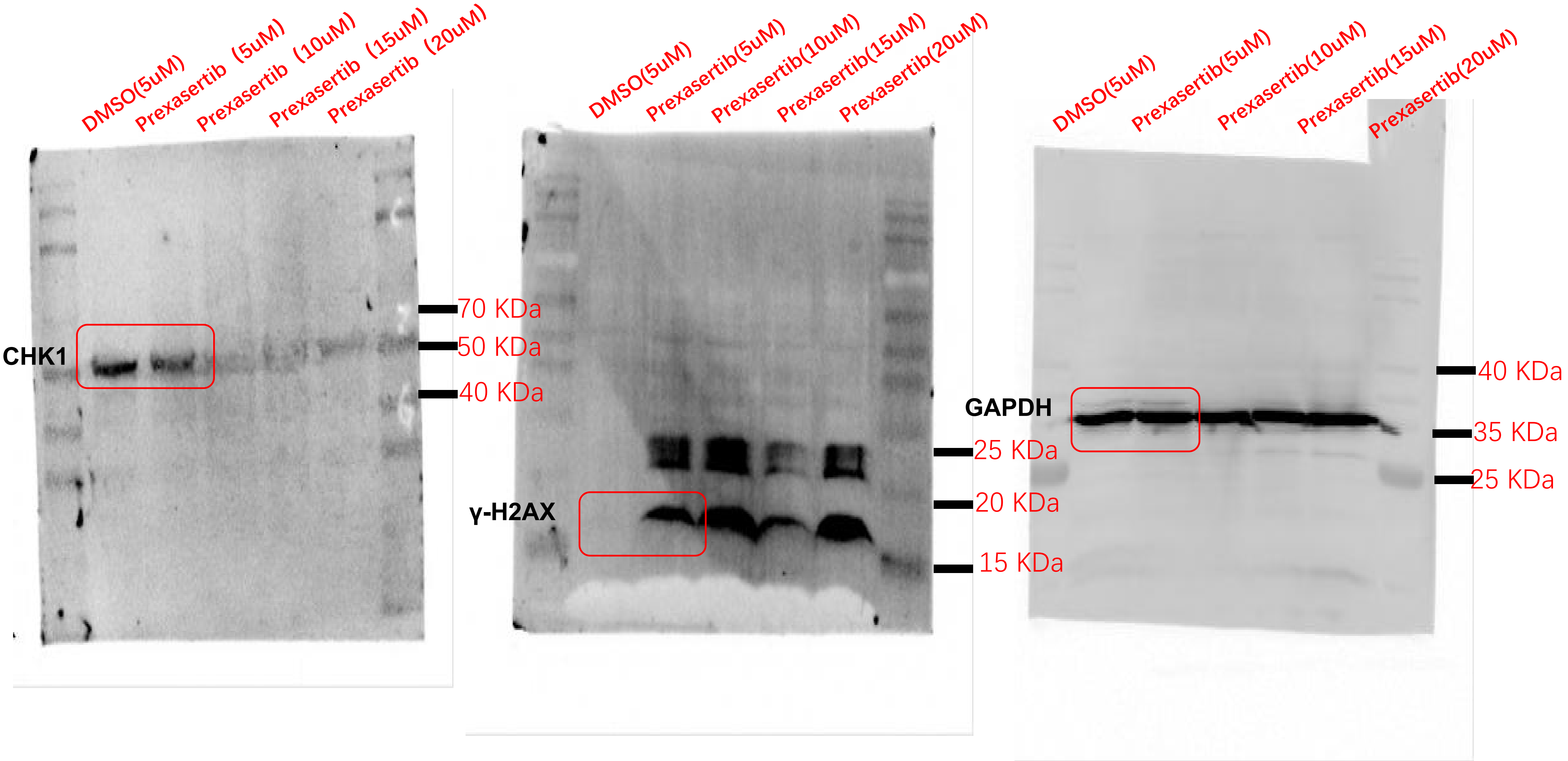

Fig.2c Huh-7 CHK1, γ-H2AX and GAPDH

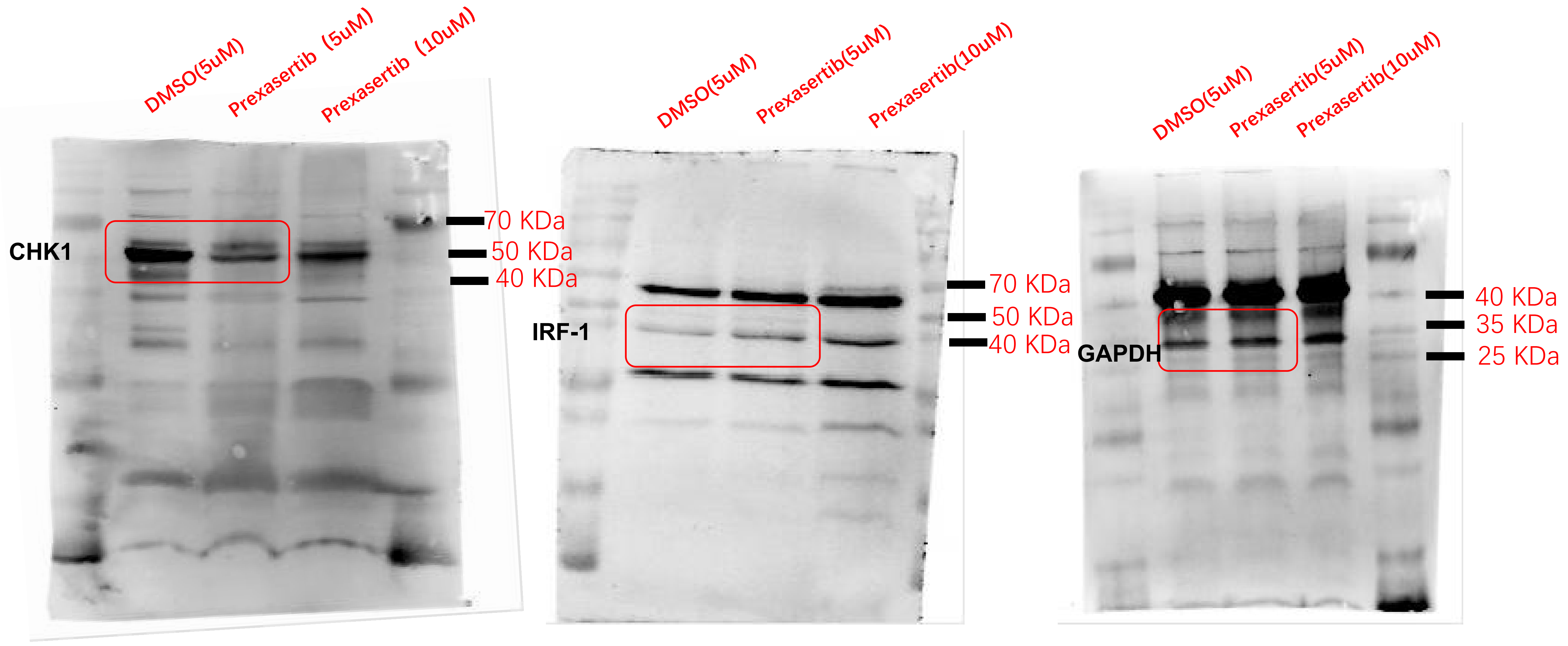

Fig.3b Huh-7 CHK1, IRF-1 and GAPDH

Fig.3d Huh-7 CHK1, IRF-1 and GAPDH

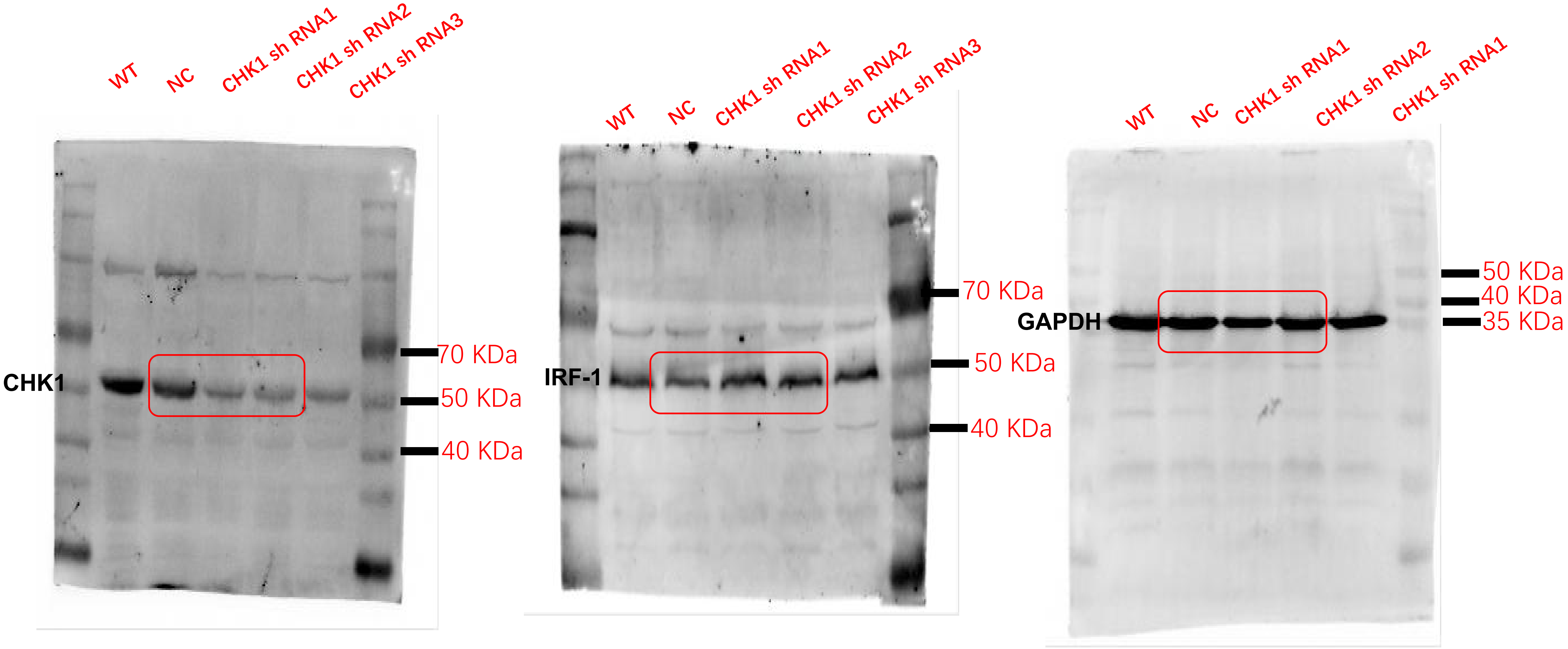

Fig.3f Huh-7 CHK1, IRF-1 and GAPDH

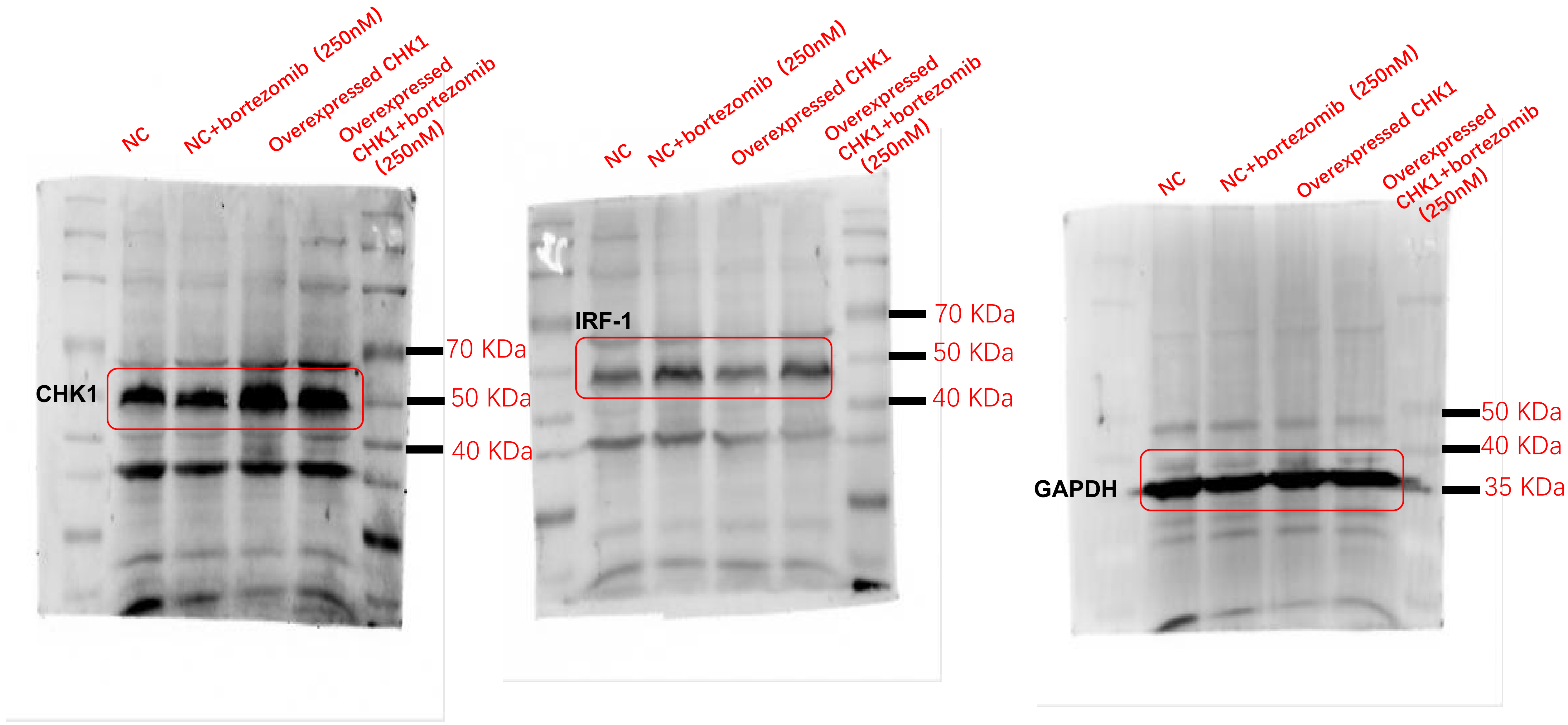

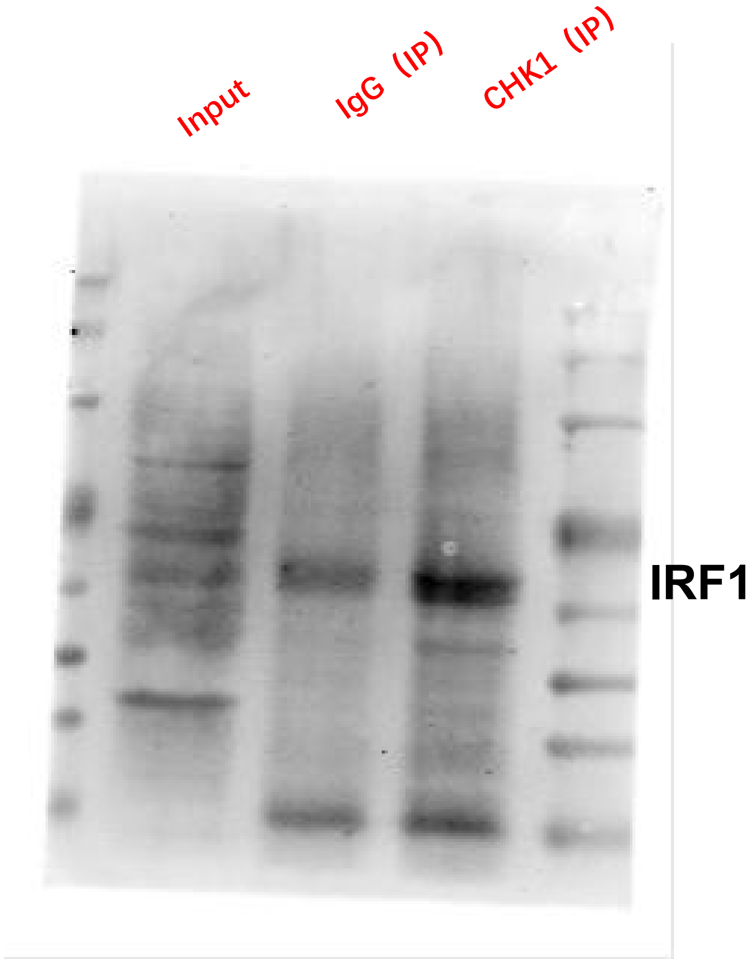

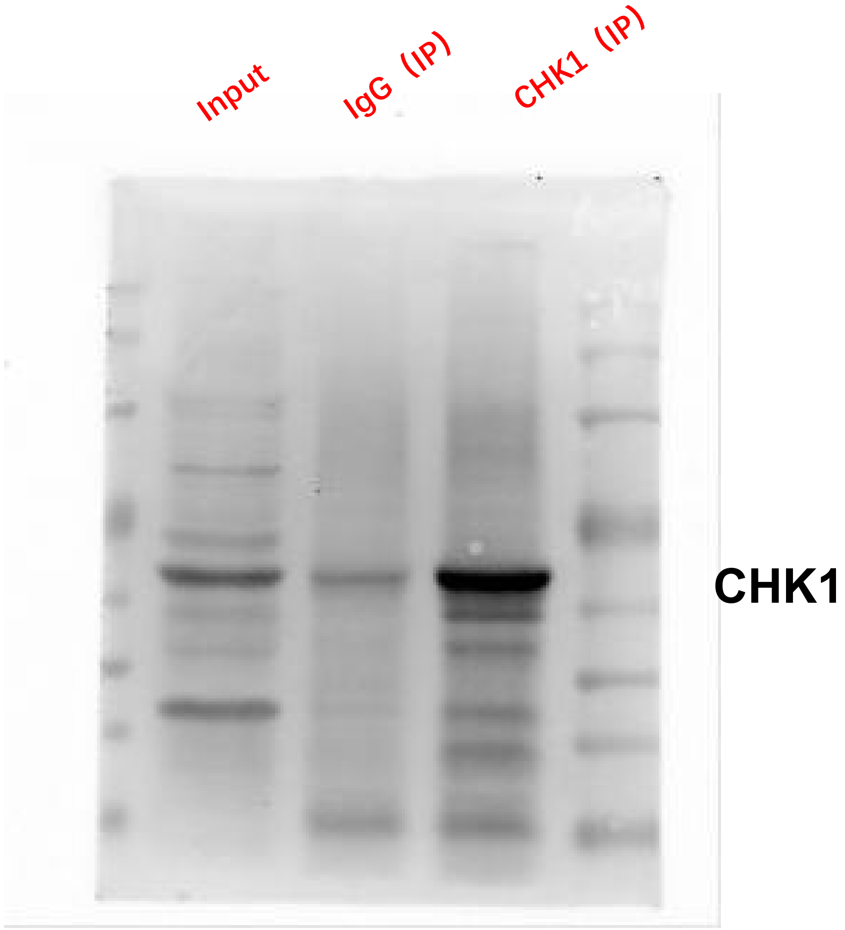

Fig.3g

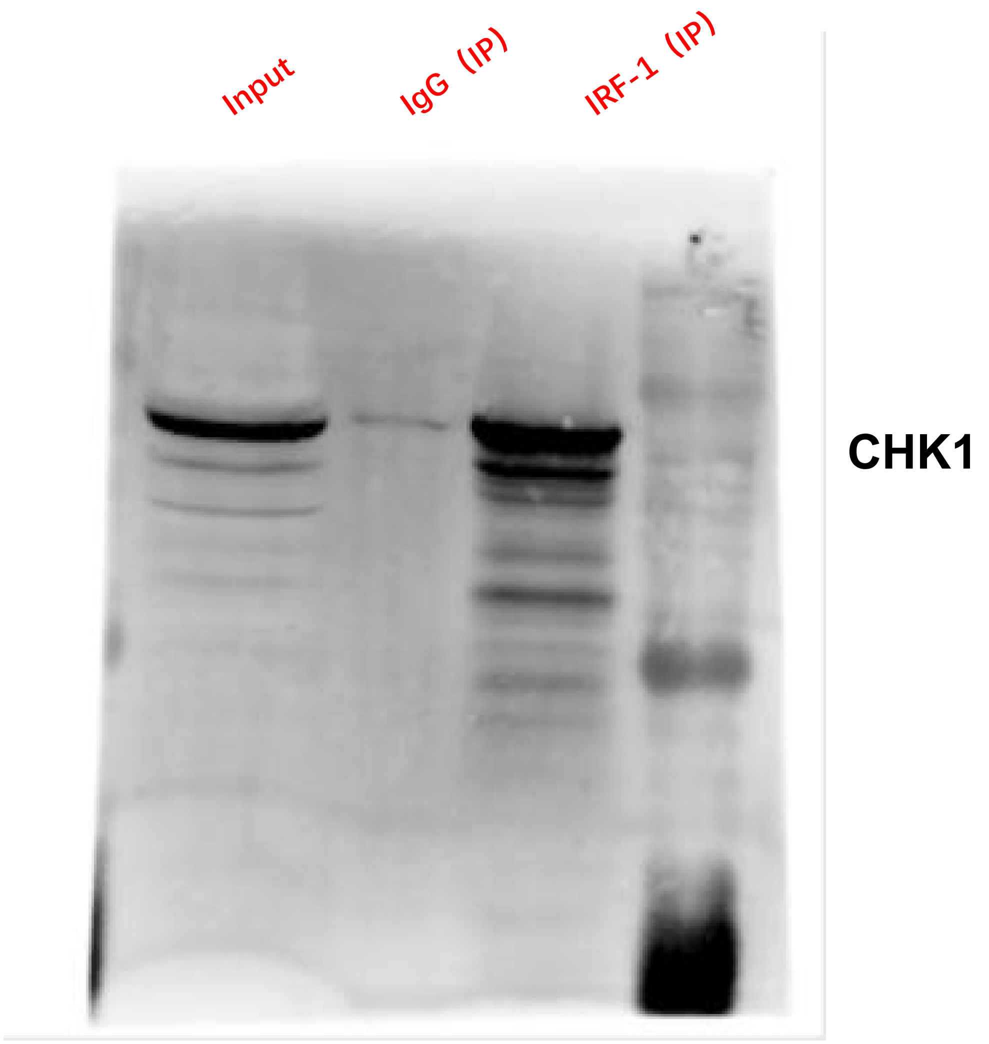

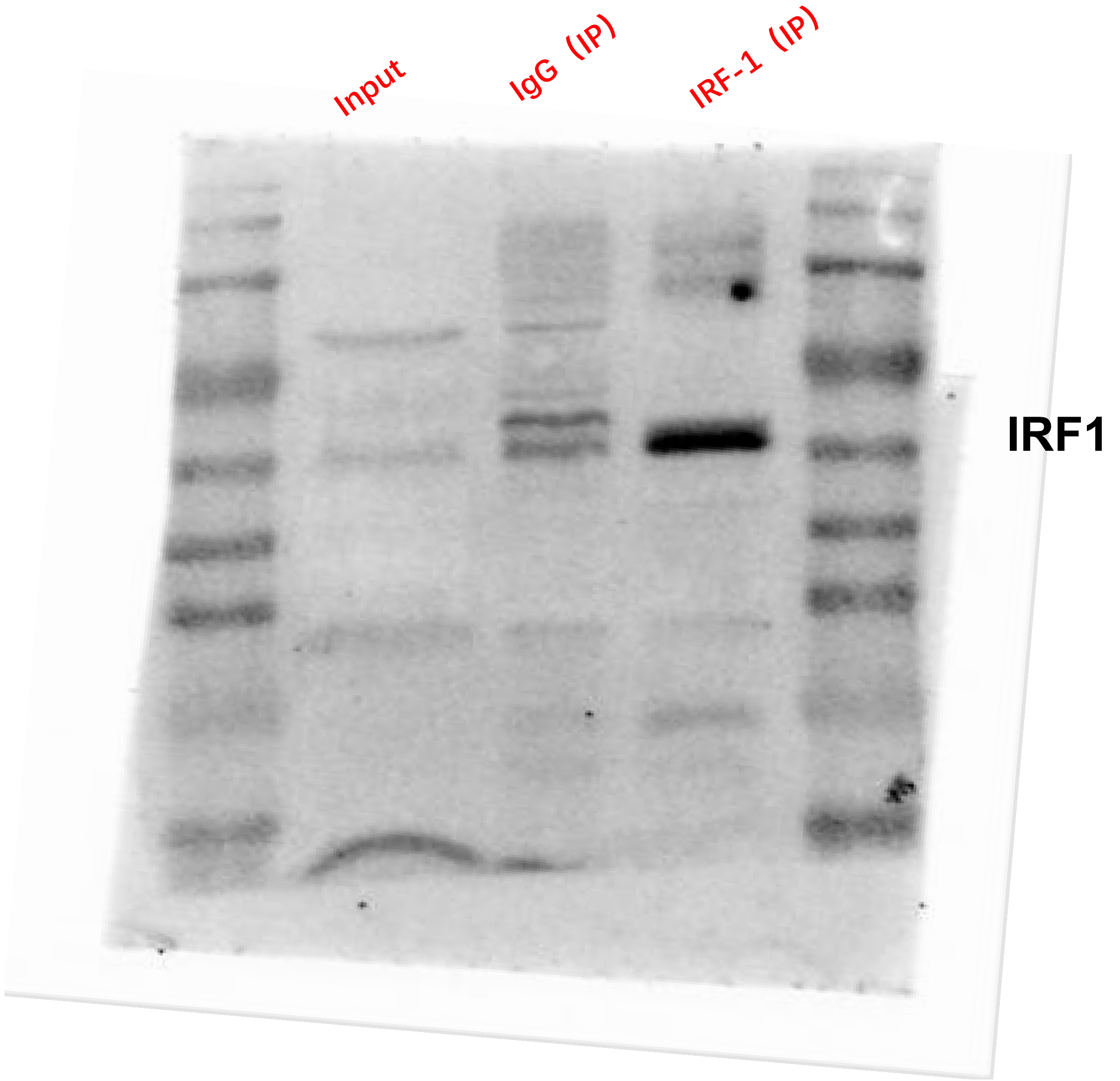

Fig.3h

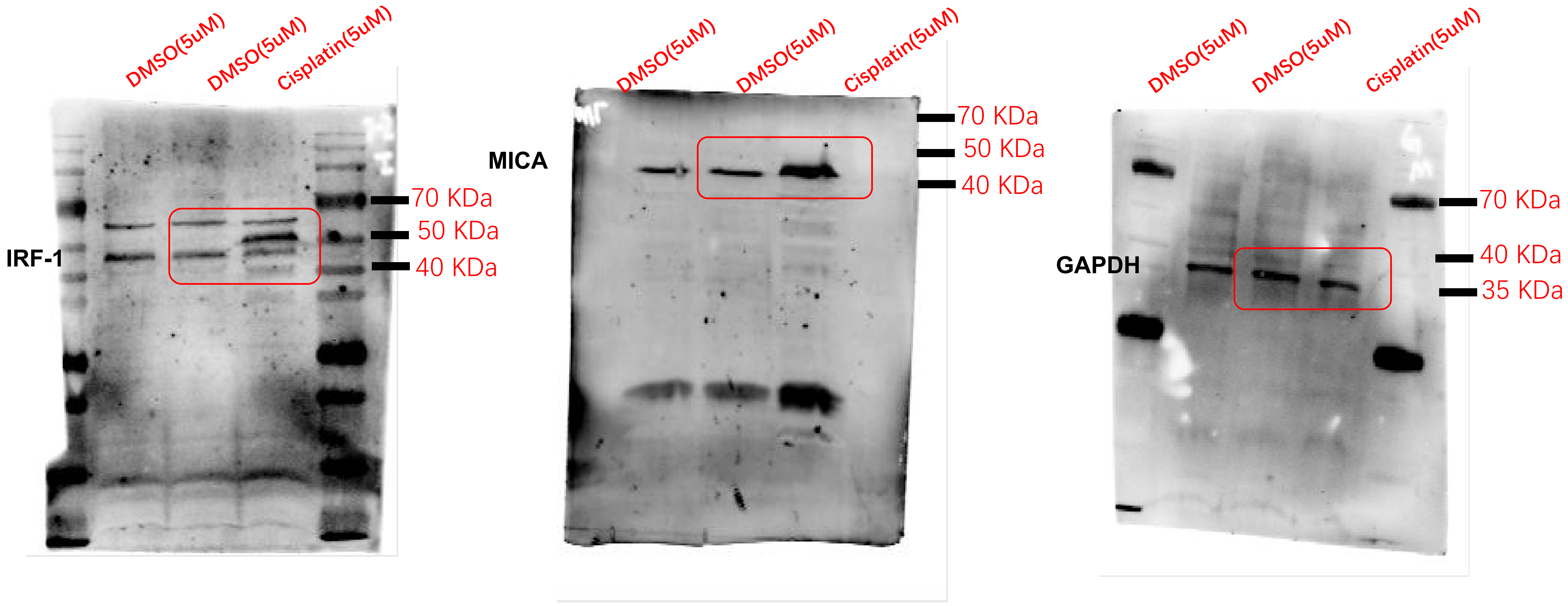

Fig.4e Huh-7 IRF-1, MICA and GAPDH

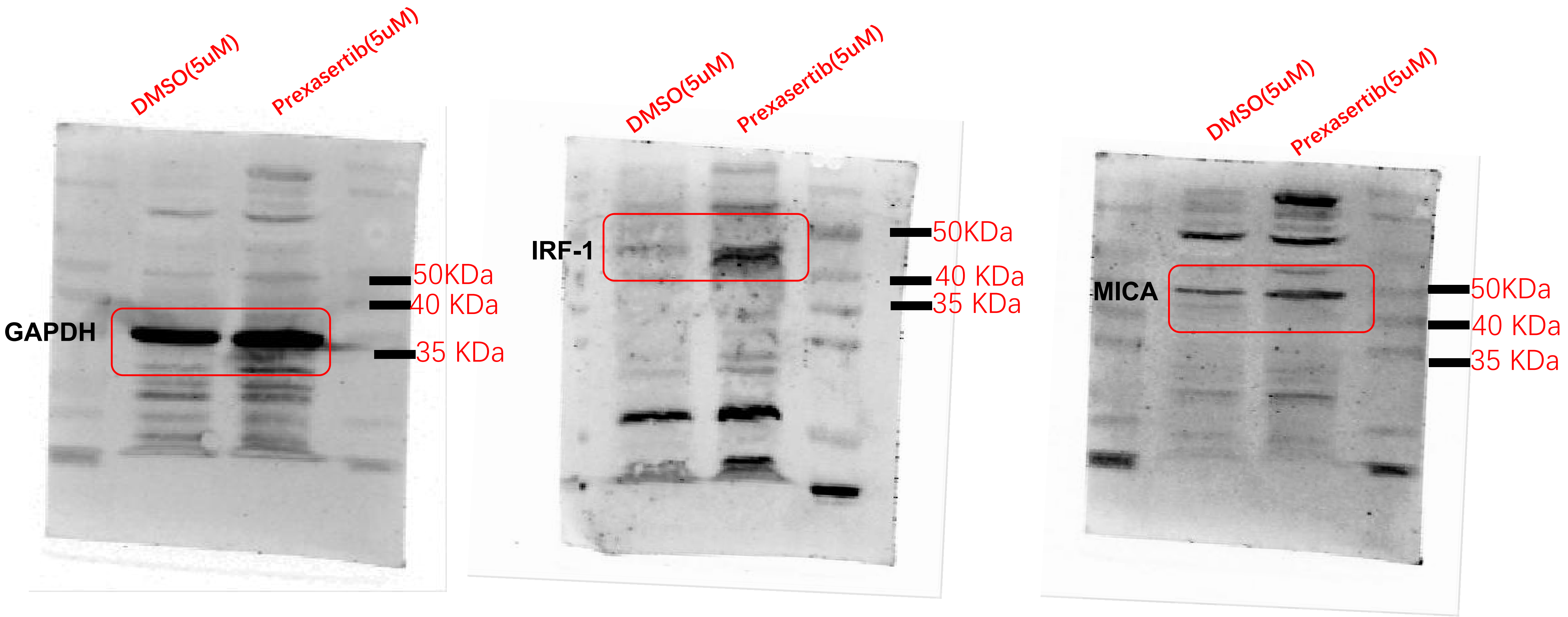

Fig.4f HepG2 GAPDH, IRF-1 and MICA

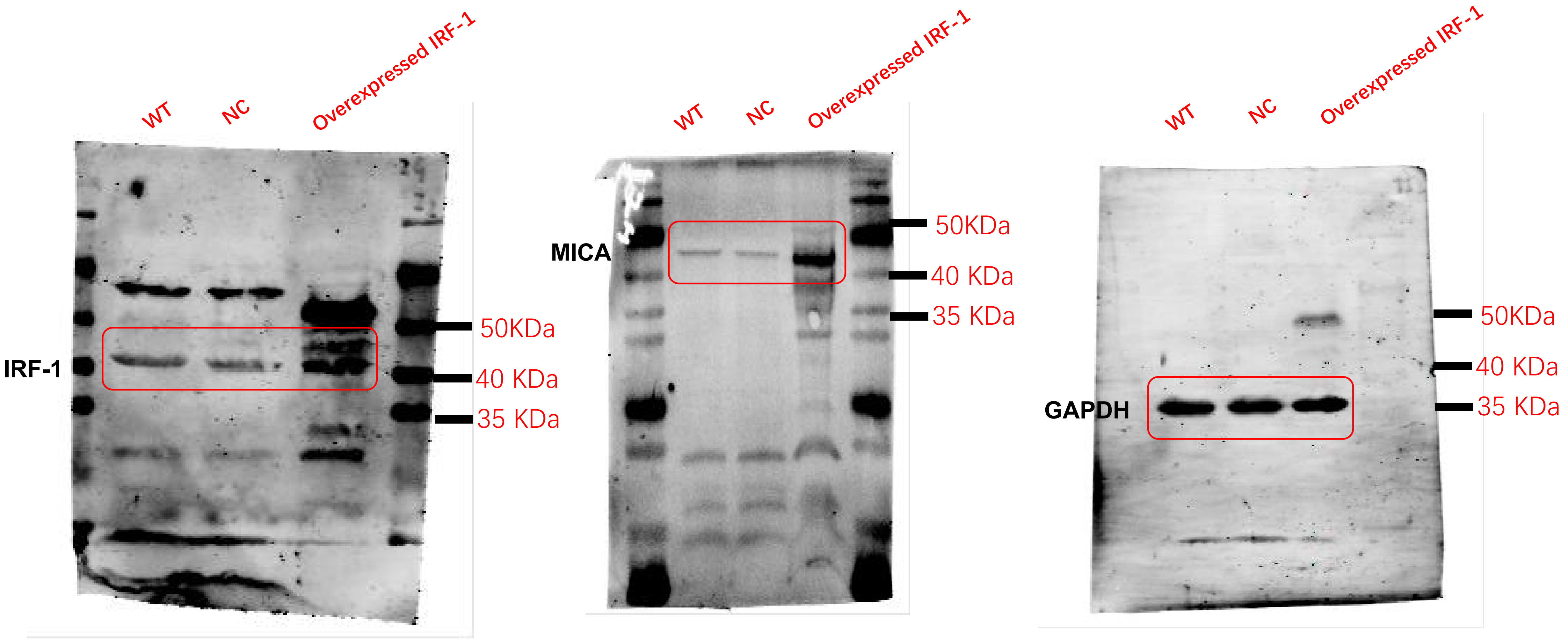

Fig.5a HepG2 IRF-1, MICA and GAPDH

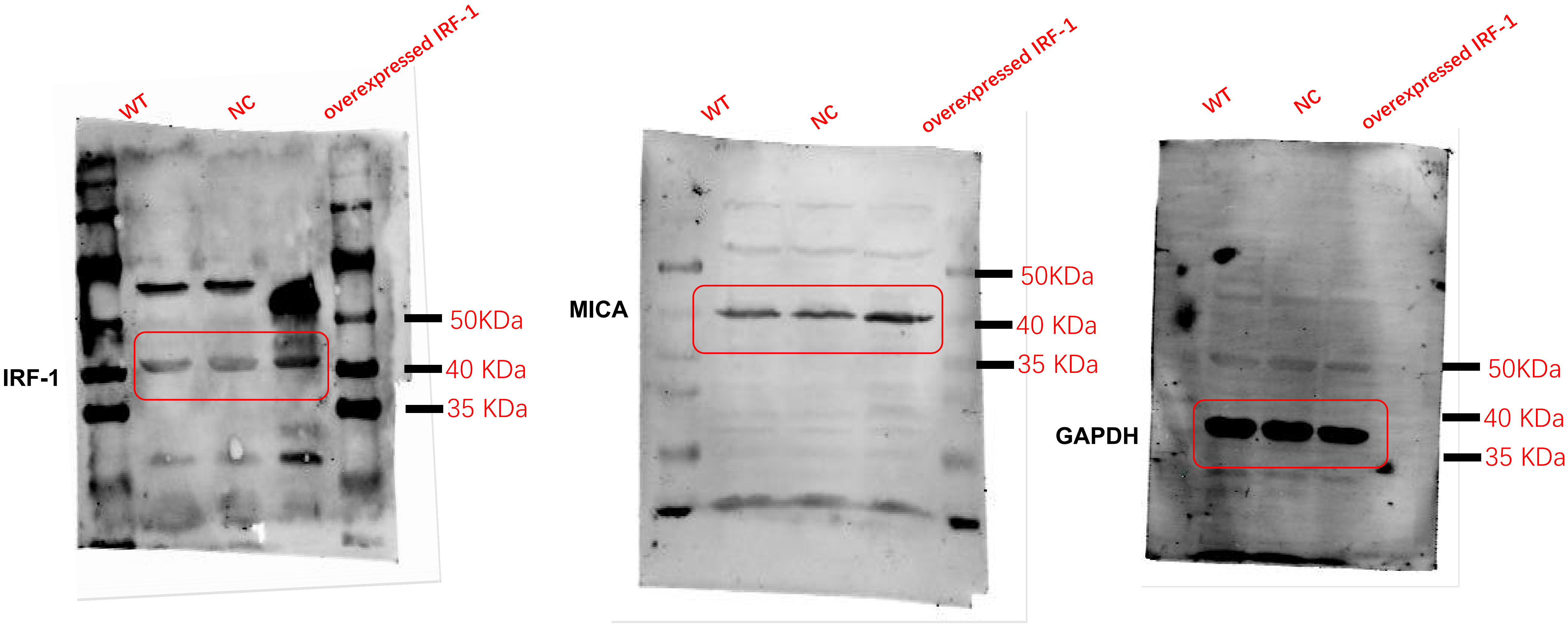

Fig.5b Huh-7 IRF-1, MICA and GAPDH

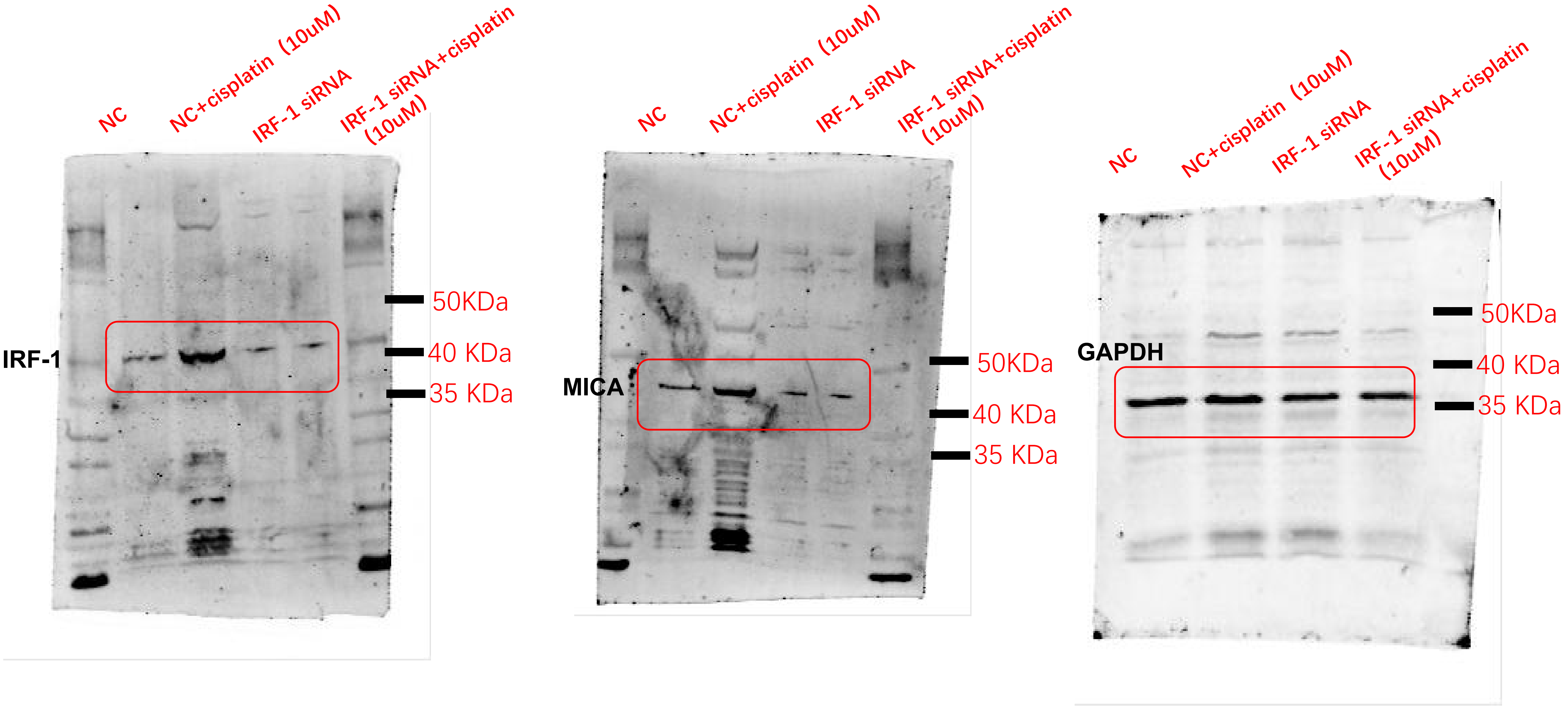

Fig.5c Huh-7 IRF-1, MICA and GAPDH
