# Supplementary material for: Inhibition of Checkpoint Kinase 1 (CHK1) Upregulates Interferon Regulatory Factor 1 (IRF1) to Promote Apoptosis and Activate Anti-Tumor Immunity via MICA in Hepatocellular Carcinoma (HCC)
Source: Cancers (Basel). 2023 Jan 30;15(3):850. doi: 10.3390/cancers15030850 (PMC9913340; doi:10.3390/cancers15030850)
Supplement: Supplementary file 1 [file cancers-15-00850-s001.zip › Supplementary Table S1.pdf]

Supplementary Table S1. The primers used in real-time RT-PCR and ChIP qPCR.

CHK1	Forward 5'-GATATGAAGCGTGCCGTAGACTGTC-3'
	Reverse 5'-GGATATTGCCTTCTCTCCTGTGACC-3'
IRF-1	Forward 5'-GGGAAATTACCTGAGGACATCA-3'
	Reverse 5'-GGCTGGACTCCAGGTTCATT-3'
MICA	Forward 5'-CACAGCGGGAATCACAGCACTC-3'
	Reverse 5'-ATAGCAGCAGCAGCAACAGCAG-3'
MICB	Forward 5'-ACCAGGATTCGCCAAGGAGAGG-3'
	Reverse 5'-GTCTGTCCGTTGACTCTGAAGCAC-3'
ULBP1	Forward 5'-CACAACCCAACCCAAGGCCATG-3'
	Reverse 5'-GCCAGCTAGAATGAAGCAGAGGAAG-3'
ULBP2	Forward 5'-GGATGGCTTGAGGACTTCTTGATGG-3'
	Reverse 5'-GATGAGGAGGCAGCAAAGGATGAG-3'
GAPDH	Forward 5'-GCACCGTCAAGGCTGAGAAC-3'
	Reverse 5'-TGGTGAAGACGCCAGTGGA-3'
β-actin	Forward 5'-TGGCACCCAGCACAATGAA-3'
	Reverse 5'-CTAAGTCATAGTCCGCCTAGAAGCA-3'
CHIP primer1	Forward 5'-ATCATTCAGTGAAGGTCACT-3'
	Reverse 5'-AGGAAGGCAGCGTCCGCCTCT-3'

CHIP primer2	Forward 5'-AACTAAGGGTGTAAATTAGGA-3'
	Reverse 5'-AAACGCTTCCTGCTCCGT-3'
CHIP primer3	Forward 5'-ACTGAAGTGGCGGGACCCTAA-3'
	Reverse 5'-ATTCCAGGATCCGAAAACGCT-3'
CHIP primer4	Forward 5'-AGAGGCGGACGCTGCCTTCCT-3'
	Reverse 5'-AGCGTGATCTGGAATCCGGCT-3'
CHIP primer5	Forward 5'-AGCTGGAGACCAGAAGCCTGA-3'
	Reverse 5'-AATTTTGCTGCCCGGAATTCA-3'
CHIP primer6	Forward 5'-AAGGCCGGGCACGGTGGTTCA-3'
	Reverse 5'-ATTCACCACCAAGCCCGTCTA-3'
CHIP Primer7	Forward 5'-AATCGCTTAAACCTTGGAGG-3'
	Reverse 5'-AAACGGCCCAGCGCGGTCGCT-3'
